# All-cause mortality in metabolically healthy individuals was not predicted by overweight and obesity

**DOI:** 10.1172/jci.insight.136982

**Published:** 2020-08-20

**Authors:** Qiuyue Tian, Anxin Wang, Yingting Zuo, Shuohua Chen, Haifeng Hou, Wei Wang, Shouling Wu, Youxin Wang

**Affiliations:** 1 Beijing Key Laboratory of Clinical Epidemiology, School of Public Health,; 2 Department of Neurology, Beijing Tiantan Hospital, and; 3 Advanced Innovation Center for Human Brain Protection, Capital Medical University, Beijing, China.; 4 Department of Cardiology, Kailuan General Hospital, North China University of Science and Technology, Tangshan, China.; 5 School of Public Health, Shandong First Medical University and Shandong Academy of Medical Sciences, Tai’an, China.; 6 School of Medical and Health Sciences, Edith Cowan University, Perth, Western Australia, Australia.

**Keywords:** Metabolism, Epidemiology, Medical statistics, Obesity

## Abstract

**BACKGROUND:**

Metabolically healthy obesity (MHO) and metabolically healthy overweight (MH-OW) have been suggested to be important and emerging phenotypes with an increased risk of cardiovascular disease (CVD). However, whether MHO and MH-OW are associated with all-cause mortality remains inconsistent.

**METHODS:**

The association of MHO and MH-OW and all-cause mortality was determined in a Chinese community-based prospective cohort study (the Kailuan study), including 93,272 adults at baseline. Data were analyzed from 2006 to 2017. Participants were categorized into 6 mutually exclusive groups, according to BMI and metabolic syndrome (MetS) status. The primary outcome was all-cause death, and accidental deaths were excluded.

**RESULTS:**

During a median follow-up of 11.04 years (interquartile range, 10.74–11.22 years), 8977 deaths occurred. Compared with healthy participants with normal BMI (MH-NW), MH-OW participants had the lowest risk of all-cause mortality (multivariate-adjusted HR [aHR], 0.926; 95% CI, 0.861–0.997), whereas there was no increased or decreased risk for MHO (aHR, 1.009; 95% CI, 0.886–1.148). Stratified analyses and sensitivity analyses further validated that there was a nonsignificant association between MHO and all-cause mortality.

**CONCLUSIONS:**

Overweight and obesity do not predict increased risk of all-cause mortality in metabolic healthy Chinese individuals.

**FUNDING:**

National Natural Science Foundation of China (NSFC; 81673247, 81872682 and 81773527), the NSFC Joint Project, and the Australian National Health and Medical Research Council (NHMRC; NSFC 81561128020-NHMRC APP1112767).

## Abstract 2

Obesity does not predict increased risk of all-cause mortality in metabolic healthy Chinese individuals.

## Introduction

Overweight and obesity have become serious public health issue in both developed and developing countries (1, 2). Studies have shown that overweight and obesity are independent risk factors for cardiovascular diseases, including coronary disease, myocardial infarction, ischemic heart disease, and malignant tumors (3–9). Two systematic reviews and meta-analyses, including at least 230 prospective studies, showed that overweight and obesity were associated with increased risk of all-cause mortality (10, 11); these findings were consistent with a study of 1.46 million White adults (12). Moreover, linear Mendelian randomization analyses have indicated that an increase of 1 unit in genetically predicted BMI gave rise to a 5%–9% increased mortality risk in overweight and obese participants (13). However, a recent 22-year cohort study found that being overweight had no effect on all-cause mortality; in particular, it was protective effect in men or people aged 30–39 years (14). A large population-based cohort study found that BMI had a J-shaped association with all-cause mortality, and the lowest risk occurred in the range 21–25 kg/m^2^ (15). A meta-analysis, including 20 prospective cohort studies, indicated that overweight and obesity were inversely associated with all-cause mortality with acute myocardial infarction history (16). Several large population meta-analyses findings, including at least 50 prospective studies, also produced the same results (17, 18). Therefore, the “obesity paradox” has been commonly identified in observational studies.

There is heterogeneity among overweight or obese individuals. Some have worse metabolic profiles and increased health risks; others have healthier metabolic profiles and decreased health risks. Combined BMI and metabolic profiles in individuals have been categorized into 6 groups: metabolically healthy normal weight (MH-NW), metabolically healthy overweight (MH-OW), metabolically healthy obesity (MHO), metabolically unhealthy normal weight (MUH-NW), metabolically unhealthy overweight (MUH-OW), and metabolically unhealthy obesity (MUO) (19–22). Thus, all obesity statuses are not equal.

In a previous study, we found that obesity was associated with myocardial infarction in a Chinese population, regardless of whether measurable metabolic abnormalities were present (21). This finding was consistent with those in several reports that aimed to identify a healthy obesity phenotype related to cardiovascular diseases (23–25). The studies about association of MH-OW or MHO phenotype with all-cause mortality risk were reported, but the results were inconsistent (26–28). Several studies have shown that MH-OW and MHO were not significantly associated with an increased risk of all-cause mortality (MH-NW as the control) (27, 29, 30). In contrast, another study demonstrated that MHO and MH-OW were not benign conditions (28). Moreover, the association of MHO or MH-OW with all-cause mortality has not been investigated in a Chinese population. In this study, we aimed to explore the association between MHO or MH-OW and all-cause mortality in the Kailuan study, a longitudinal study with 101,510 participants and more than 10 years of follow-up.

## Results

**Figure 1:**
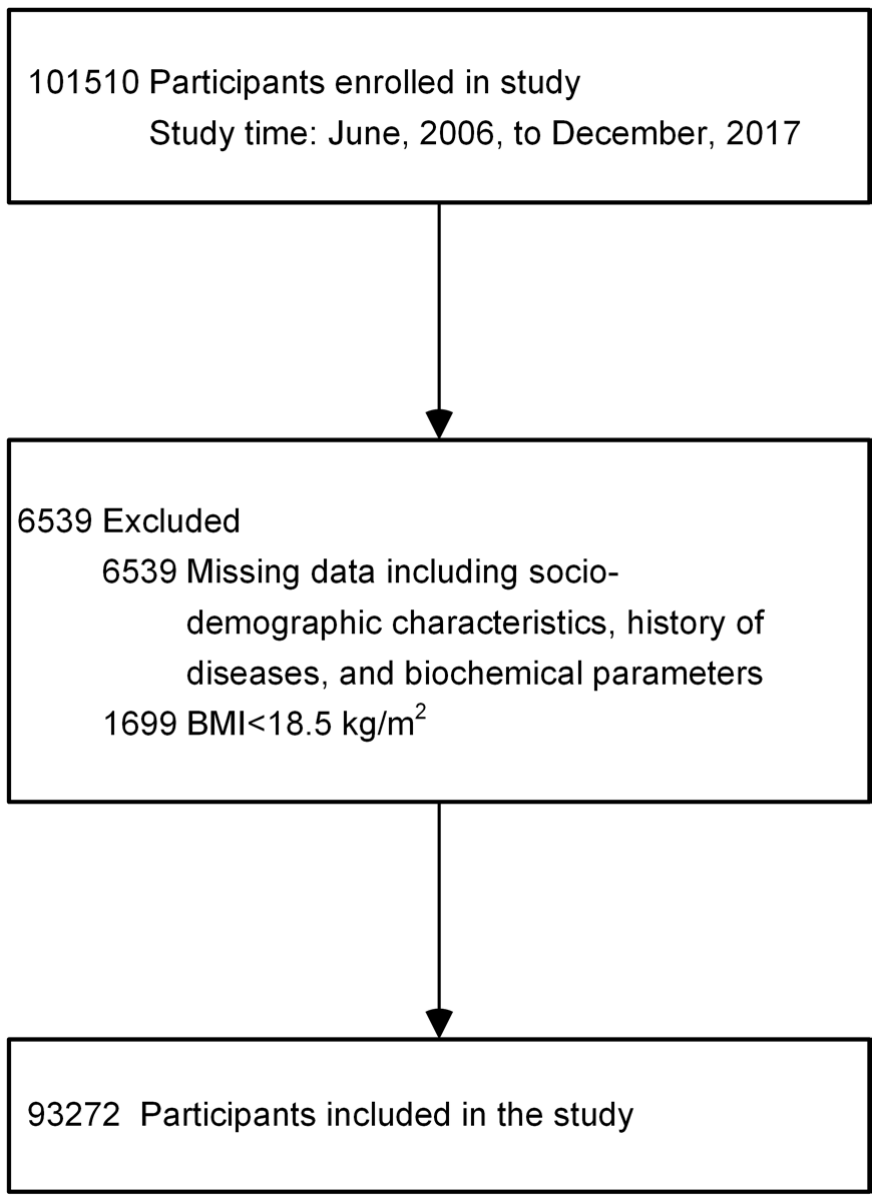
The flow diagram of study participants.

Participants with missing data (*n* = 6539) or BMIs of less than 18.5 kg/m^2^ (*n* = 1699) were excluded. Some individuals met more than 1 exclusion criterion. A total of 93,272 eligible participants were finally included in the analyses (Figure 1).

Among the 93,272 eligible participants, 80,569 (86.38%) were metabolically healthy. MH-OW and MHO statuses represented 36.17% (*n* = 33,736) and 14.22% (*n* = 13,266) of the total samples, respectively. The demographic and biochemical characteristics of the participants are presented in Table 1. Compared with MH-NW individuals, MH-OW and MHO individuals had a history of disease and a higher proportion of older individuals, men, and drinkers. The levels of HDL cholesterol (HDL-C) and education in the MH-OW and MHO groups were significantly lower than those in the MH-NW groups. In addition, higher blood pressure, triglyceride (TG), waist circumference (WC), LDL, and salt intake levels were also found in the MH-OW and MHO groups (Table 1). In addition, MH-OW and MHO individuals had a higher proportion of abnormal measures in other indices of disease, including LDL cholesterol (LDL-C), fasting blood glucose (FBG), TG, and HDL-C, compared with those in MH-NW individuals (*P* < 0.001) (Table 1).

**Figure 2:**
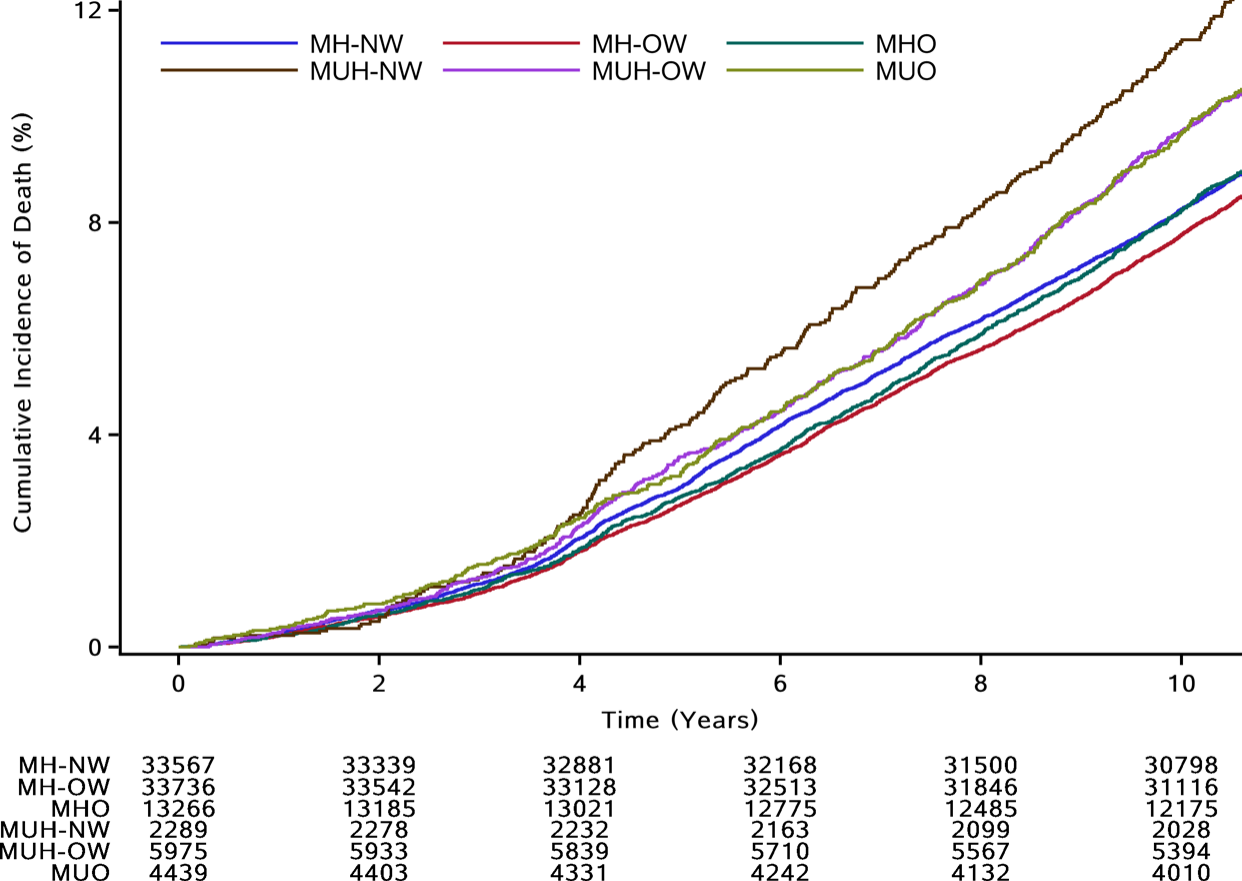
The cumulative incidence of death according to the BMI-MetS phenotypes. MetS, metabolic syndrome; MH-NW, metabolically healthy normal weight; MUH-NW, metabolically unhealthy normal weight; MH-OW, metabolically healthy overweight; MUH-OW, metabolically unhealthy overweight; MHO, metabolically healthy obesity; MUO, metabolically healthy obesity.

**Figure 3:**
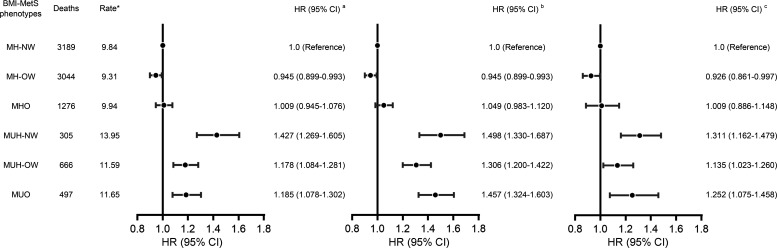
Association of BMI-MetS phenotypes with all-cause mortality risk. Multivariate cox regression analysis was used to evaluate the association of all-cause mortality risk with BMI-MetS phenotypes, adjusting for potential confounding factors (*n* = 93,272). HR calculated by univariate cox regression (left), HR calculated by cox regression adjusting for age and sex (middle), and HR calculated by cox regression further adjusted for smoking, drinking, education, BMI index, income, exercise, salt intake, and history of disease (hyperlipidemia, hypertension, diabetes, myocardial infarction, and stroke) (right) are shown. *Per 1,000 person-years. MH-NW, metabolically healthy normal weight; MUH-NW, metabolically unhealthy normal weight; MH-OW, metabolically healthy overweight; MUH-OW, metabolically unhealthy overweight; MHO, metabolically healthy obesity; MUO, metabolically healthy obesity; MetS, metabolic syndrome.

After a median follow-up period of 11.04 years (interquartile range, 10.74–11.22 years), 8977 deaths occurred. The incidences of all-cause death per 1000 person-years were 9.84 in the MH-NW group, 9.31 in the MH-OW group, 9.94 in the MHO group, 13.95 in the MUH-NW group, 11.59 in the MUH-OW group, and 11.65 in the MUO group. As shown in Figure 2, all-cause mortality was highest in the MUH-NW group and the lowest in the MH-OW group. In the crude Cox model, compared with participants in the MH-NW group, participants were at a 5.5% lower risk (HR, 0.945; 95% CI, 0.899–0.993) in the MH-OW group, at no risk (HR, 1.009; 95% CI, 0.945–1.076) in the MHO group, at a 42.7% higher risk (HR, 1.427; 95% CI, 1.269–1.605) in the MUH-NW group, at a 17.8% higher risk (HR, 1.178; 95% CI, 1.084–1.281) in the MUH-OW group, and at a 18.5% higher risk (HR, 1.185; 95% CI, 1.078–1.302) in the MUO group for all-cause mortality (Figure 3). After adjustment for sex, age, WC, history of disease, socioeconomic status, lifestyle factors, and dyslipidemia, the HRs for all-cause mortality were 0.926 (95% CI, 0.861–0.997) in the MH-OW group, 1.009 (95% CI, 0.886–1.148) in the MHO group, 1.311 (95% CI, 1.162–1.479) in the MUH-NW group, 1.135 (95% CI, 1.023–1.260) in the MUH-OW group, and 1.252 (95% CI, 1.075–1.458) in the MUO group, compared with the MH-NW group (Figure 3).

**Figure 4:**
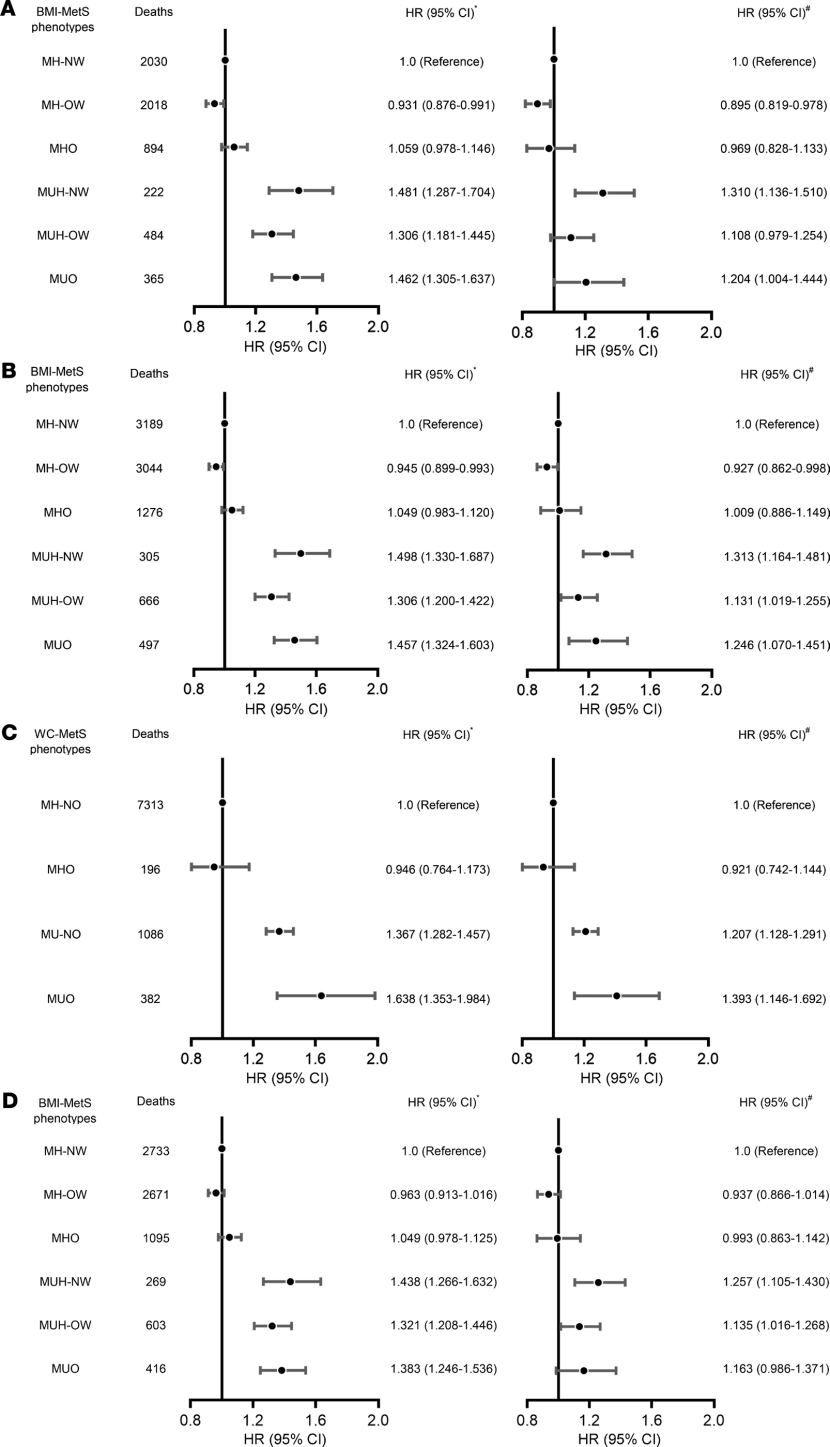
The sensitivity analyses of the association of all-cause mortality risk with BMI-MetS phenotypes. The association of all-cause mortality risk with BMI-MetS phenotypes excluding current smokers (**A**), excluding dead participants during the first 2 years (**B**), using WC instead of BMI and modifying the definition of MetS (≥2 among the 4 components excluding the WC criteria) (**C**), retaining participants aged 50–85 years (**D**). *n* = 61,002 (**A**); *n* = 93,272 (**B**); *n* = 93,272 (**C**); *n* = 52,776 (**D**). Multivariate cox regression analysis was used to evaluate the association of all-cause mortality risk with BMI-MetS phenotypes, adjusting for potential confounding factors. The asterisk indicates HR calculated by cox regression adjusting for age and sex, and the pound sign indicates HR calculated by cox regression further adjusting for smoking, drinking, education, BMI index, income, exercise, salt intake, dyslipidemia, and history of disease (hypertension, hyperlipidemia, diabetes, myocardial infarction, and stroke). MH-NW, metabolically healthy normal weight; MUH-NW, metabolically unhealthy normal weight; MH-OW, metabolically healthy overweight; MUH-OW, metabolically unhealthy overweight; MHO, metabolically healthy obesity; MUO, metabolically healthy obesity; MH-NO, metabolically healthy normal waist circumference; MU-NO, metabolically unhealthy normal waist circumference; MetS, metabolic syndrome; WC, waist circumference.

In the sensitivity analyses, we first carried out the main analysis among participants with no smoking habit at baseline, and similar results were obtained (Figure 4). The adjusted HRs were 0.895 (95% CI, 0.819–0.978) in the MH-OW group, 0.969 (95% CI, 0.828–1.133) in the MHO group, and 1.310 (95% CI, 1.136–1.510) in the MUH-NW, compared with the MH-NW group (Figure 4A). The sensitivity analyses also showed similar results after the exclusion of participants who died during the first 2 years of follow-up (Figure 4B). Furthermore, we redefined obesity and metabolic syndrome (MetS) and obtained similar results. Compared with MH-NO individuals, MHO individuals had no significantly increased risk of all-cause mortality (HR, 0.921, 95% CI, 0.742–1.144), whereas MU-NO and MUO individuals had a significantly increased risk of all-cause mortality (MU-NO, HR, 1.207, 95% CI 1.128–1.291; MUO, HR, 1.393, 95% CI 1.146–1.692, respectively) after controlling for all confounding factors (Figure 4C). In addition, the results showed that, for participants aged 50–85 years, adjusted HRs were 0.937 (95% CI, 0.866–1.014) in the MH-OW group, 0.993 (95% CI, 0.863–1.142) in the MHO group, and 1.257 (95% CI, 1.105–1.430) in the MUH-NW, compared with the MH-NW group (Figure 4D).

In the stratified analyses, compared with that in the MH-NW group, MH-OW was associated with significantly decreased risk of all-cause mortality in women (HR, 0.732, 95% CI, 0.544–0.986), but there was no increased or decreased risk in men (HR, 0.938, 95% CI, 0.869–1.012). The association was significant in participants with a baseline age <65 years (HR, 0.895, 95% CI, 0.807–0.993) but not in those with baseline age ≥65 years (HR, 0.951, 95% CI, 0.857–1.055) (Table 2). Similarly, MHO was not significantly associated with an increased risk of mortality in each stratum, and MUH remained the highest-risk phenotype for all-cause mortality. There were significant interactions of age (<65 years old, ≥65 years old) and sex in relationship BMI-MetS phenotypes with all-cause mortality (*P* interaction < 0.01 for both), but no interactions were found for smoking status, drinking status, or physical activity (*P* interaction > 0.05 for all) (Table 2).

## Discussion

In a prospective cohort study with a median follow-up of 11 years, we found that MH-OW participants had the lowest risk of all-cause mortality (HR, 0.926, 95% CI, 0.861–0.997) and participants MHO participants did not have an increased risk of all-cause mortality (HR, 1.009, 95% CI, 0.886–1.148), while MUH-NW participants had the highest risk of all-cause mortality (HR, 1.311, 95% CI, 1.162–1.479) compared with the MH-NW participants, after adjusting for sex, age, WC, history of disease, socioeconomic status, lifestyle factors, and dyslipidemia. The sensitivity and stratification analyses further validated these findings. To our knowledge this is the first large population study to demonstrate that overweight and obesity do not predict an increased risk of all-cause mortality in a metabolically healthy Chinese population.

The association between MHO and all-cause mortality has been widely investigated, but the findings remain inconsistent. A systemic review and meta-analysis, including 11 prospective studies from Europe, North America, and Asia (published from 1950 to June 5, 2013), indicated that MHO was not significantly associated with all-cause mortality and/or cardiovascular events (relative risk [RR]: 1.07, 95% CI, 0.92–1.25) but was significantly associated when only studies with at least 10 years of follow-up were included (RR, 1.24, 95% CI, 1.02–1.55) (31). Another systematic review and meta-analysis (published up until September 30, 2015) demonstrated that MHO was not associated with increased all-cause mortality risk (HR, 1.07; 95% CI, 0.92–1.25) (19). Recently, most studies have not favored the association between the MHO phenotype and an increased risk of all-cause mortality. A cohort study (54,089 participants, 12.8 years of follow-up) combining 5 cohort studies (Aerobics Center Longitudinal Study [ACLS], Coronary Artery Risk Development in Young Adults [CARDIA], Multi-Ethnic Study of Atherosclerosis [MESA], National Health and Nutrition Examination Survey [NHANES III], and National Health and Nutrition Examination Survey Continuous [NHANES Continuous]) showed that obesity without other metabolic risk factors was not associated with an increased risk of all-cause mortality compared with lean healthy individuals (HR, 1.10, 95% CI 0.8–1.6) (32). The English Longitudinal Study of Ageing (5427 participants, 8 years of follow-up) also indicated that there was no significant association between MHO and all-cause mortality (HR, 1.14, 95% CI 0.83–1.52) (33). Another cohort study carried out in the United Kingdom (22,203 participants, follow-up 7 years) also revealed that the MHO phenotype (HR, 0.91; 95% CI, 0.64–1.29) did not increase all-cause mortality risk compared with metabolically healthy individuals without obesity (27). A prospective cohort study in Finland (2185 men, 26 years of follow-up) demonstrated that MH-OW/obese men were not at increased risk of sudden cardiac death (HR, 0.95; 95% CI, 0.40–2.24) compared with the MH-NW group (34). Consistent with these recent studies, the present study (93,272 participants, 11 years of follow-up) verified no significant association between MHO and all-cause mortality in a Chinese population, suggesting that baseline obesity without MetS does not have adverse effects to all-cause mortality.

Contrary to the approximately well-defined association between MHO and all-cause mortality, the association between MH-OW and all-cause mortality is more complex. Previously described systemic review and meta-analysis has indicated that MH-OW was not significantly associated with all-cause mortality and/or cardiovascular events, in all studies (RR, 1.10, 95% CI, 0.90–1.24) or only in studies with at least 10 years of follow-up (RR, 1.21, 95% CI, 0.91–1.61) (31). Additionally, the cohort study (54,089 participants, 12.8 years of follow-up) combining 5 cohort studies (ACLS, CARDIA, MESA, NHANES, and NHANES Continuous) showed that overweight without other metabolic risk factors was not associated with an increased risk of all-cause mortality compared with lean healthy individuals (HR, 0.95, 95% CI 0.7–1.2) (32). Most studies of the association between MH-OW and all-cause mortality demonstrated a negative relationship. The Reasons for Geographic and Racial Dereferences in Stroke (REGARDS) cohort study (22,514 participants, 6.5 years of follow-up) demonstrated that the MH-OW phenotype (HR, 0.79; 95% CI, 0.63–0.98) was associated with a decreased risk of cancer mortality (35). In contrast to these studies, we demonstrated that the MH-OW phenotype was associated with a decreased risk of all-cause mortality (HR, 0.926; 95% CI, 0.861–0.997), suggesting that the MH-OW phenotype might be an independent protective factor for all-cause mortality.

An unusual, but understandable, finding was that participants with the MUH-NW phenotype were at the highest risk for all-cause mortality among 6 metabolic phenotypes (HR, 1.311, 95% CI, 1.162–1.479) in the present study. Consistent with our findings, several studies have shown that MUH-NW individuals were at increased risk for future cardiometabolic disease, including atrial fibrillation (26), hypertension (36), kidney disease (37), and death (27) compared with the MH-NW individuals. Similarly, in a pooled analysis of 8 studies, the MUH-NW group (RR, 3.14; 95% CI, 2.36–3.93) had the highest risk for all-cause mortality compared with individuals with the other 5 metabolic phenotypes (19). Consistent with these findings, we observed that there was a highest risk of all-cause mortality in participants with the MUH-NW phenotype than in those with other phenotypes (HR, 1.311, 95% CI, 1.162–1.479). This counterintuitive and perhaps unexpected result might be explained by the fact that the MUH-NW phenotype represents the most severe subtype along the phenotypic spectrum of individuals genetically predisposed to cardiovascular events or death (19). Genetic analyses supported the notion that metabolically unhealthy phenotypes might be associated with body fat distribution patterns that favor visceral and ectopic fat accumulation over fat deposition in the periphery (38, 39). Furthermore, MUH-NW is most strongly characterized by a low percentage of gluteofemoral and leg fat mass (40). On the other hand, MUH-NW participants might have other undefined abnormalities (19, 41–43) or metabolic abnormalities, resulting in fat distribution changes (44, 45), which might contribute to this adverse phenotype. In addition, the finding is supported by the observation that MUH-NW groups had high percentage of history of diabetes compared with other group (Table 1). Consequently, substantial attention should be given to individuals with metabolically unhealthy status, despite normal weight.

MH-OW was found to be the healthiest metabolic phenotype, which is the most important finding of the present study. This large-scale prospective study, including approximately 100,000 participants who were followed-up for more than 10 years, might have resulted in the robust findings. Second, we first verified that MHO or MH-OW did not increase the risk for all-cause mortality in a Chinese population. At this point, the present study supported the concept that “all obesity is not created equally.” However, considering our previous finding that obesity was associated with a higher risk of myocardial infarction, even without measurable metabolic abnormalities (21), whether participants with the MHO or MH-OW phenotype should reduce their body weight needs further consideration.

Apart from its strengths, several limitations should be addressed. First, there is no universally accepted definition for metabolic health, such as, 0 or 1 cardiometabolic abnormalities, fewer than 2 signs of metabolic components, or other criteria (46–48). Many previous studies have used the International Diabetes Federation criteria to define metabolic health as the presence of less than 2 MetS components (21, 26, 49). Therefore, we also adopted the above criteria to define metabolic health. Second, metabolic health status might change over time, specifically among individuals with obesity (50, 51); therefore, the baseline status did not represent actual exposure in a longitudinal study. Third, although a range of potential confounding factors was adjusted in the multivariate analysis, the bias resulting from unmeasured and residual confounding factors could not be completely avoided. Finally, the unbalanced sex ratio (Table 1) might restrict the generalization of the present findings. However, the consistencies among sensitivity and stratified analyses might minimize the limitation.

In brief, the present study shows that overweight and obesity do not predict increased risk of all-cause mortality in metabolic healthy Chinese individuals. Metabolic healthy overweight is the healthiest phenotype when only all-cause mortality was taken into account.

## Methods

*Studypopulation*. The Kailuan study is an ongoing prospective cohort study in Tangshan, China. This study was designed to investigate risk factors for chronic diseases (such as stroke, myocardial infarction, cancer, etc.). From June 2006 to October 2007, a total of 101,510 adults (81,110 men and 20,400 women) aged 18–98 years were enrolled to participate in a routine medical examinations, which included physical examination, routine blood, urine, and biochemical tests every 2 years at 11 hospitals affiliated with the Kailuan community (52–54). In this analysis, we included participants from the Kailuan study, excluding participants with missing data for biochemical parameters, sociodemographic characteristics, history of disease or current use of medication (hypertension, diabetes, stroke, and myocardial infarction) and if their BMI was less than 18.5 kg/m^2^ at baseline.

### Exposure factors.

BMI was calculated as weight in kilograms divided by height in meters squared, and participants were categorized into normal (18.50 kg/m^2^ ≤ BMI < 24.00 kg/m^2^), overweight (24.00 kg/m^2^ ≤ BMI < 28.00 kg/m^2^), or obese (BMI ≥ 28.00 kg/m^2^) groups according to Chinese-specific criteria (55). Based on the modified International Diabetes Federation criteria for the Asian population, MetS was defined as the presence of 3 or more abnormal components (WC ≥80 cm in women and ≥90 cm in men; TG ≥1.70 mmol/L or current use of lipid-lowering agents; diastolic blood pressure ≥85 mmHg, systolic blood pressure ≥130 mmHg, or self-reported history of hypertension or current use of blood pressure medication; FGB level ≥5.60 mmol/L, current use of glucose-lowering agents or self-reported history of diabetes; and HDL-C <1.03 mmol/L for men and <1.30 mmol/L for women or current use of lipid-lowering agents) (56). Metabolically healthy (MH) was defined as the presence of 2 or less abnormal components, while metabolically unhealthy (MUH) was defined as the presence of 3 or more abnormal components. Combined with BMI category (normal weight, overweight, and obesity), metabolic healthy participants were divided into 3 phenotypes, MH-NW, MH-OW, and MHO, and metabolic unhealthy participants were divided as well, MUH-NW, MUH-OW, and MUO (21, 46).

### Covariables.

Face-to-face questionnaire interviews and clinical examinations were conducted by well-trained medical staff following a standard protocol to collect information on sociodemographic characteristics, lifestyle factors, and medical history (57). Smoking and drinking status were divided into 3 categories: never, former, and current (21, 52). Physical activity was evaluated with regard to the frequency of physical activity, including inactive; moderately active, 1–2 times/week; and vigorously active, ≥3 times/week and ≥30 minutes (53). In addition, levels of FBG, TG, and HDL-C were measured using an autoanalyzer (Hitachi 747) at the central laboratory of Kailuan General Hospital (58).

### Follow-up and outcome.

All participants were followed by face-to-face interviews at every 2-year routine medical examination until December 31, 2017, or until death. The follow-ups were performed by hospital physicians, research physicians, and research nurses, who were blinded to the baseline data. For the participants without face-to-face follow-up, the follow-up information was collected by referring to death certificates from provincial vital statistics offices, discharge summaries from the 11 hospitals, or medical records from medical insurance (59).

We used all-cause death as the primary outcome. Considering unnatural death, we excluded the accidental deaths, which were transport-related accidents, violence, falling, natural hazard, medical malpractice, and food poisoning. Deaths were assessed using family report, death certificates from provincial vital statistics offices, and medical records from medical insurance or hospitals (52).

### Statistics.

The baseline characteristics of participants are presented as mean ± standard deviation or median with interquartile range for continuous variables and percentage for categorical variables. χ^2^ tests were used for the comparisons of categorical variables. The analysis of variance or Kruskal-Wallis tests were used for continuous variables. Person-years were calculated from the date of baseline examination to the date of death or the end of follow-up (December 31, 2017), whichever came first. The cumulative mortality among 6 phenotype groups was estimated using the Kaplan-Meier method and compared by log-rank tests. The Sidak method was used to adjust *P* values in the multiple comparisons (21).

Cox proportional hazards regression was used to estimate HRs and 95% CIs for the association between the 6 BMI-MetS groups and all-cause mortality risk. The proportional hazards assumption was tested by the Schoenfeld residuals (21), and no violation was found. We fitted 3 Cox proportional hazard models. Model 1 was a crude model without adjusted covariates. Model 2 was adjusted for age and sex. Model 3 was further adjusted for smoking status, drinking status, educational level, family per-member monthly income, physical activity, salt intake, dyslipidemia, and history of disease.

To test the robustness of the main results, we conducted 4 sensitivity analyses in model 1 and model 3. We excluded participants who were current smokers at baseline or died during the first 2 years of follow-up. In addition, we defined obesity using WC instead of BMI and defined MetS as having 2 or more of 4 metabolic components (excluding WC criteria). Participants were classified into the following 4 groups: no obesity (WC <80 cm in women and <90 cm in men) without MetS (MH-NO) or with MetS (MU-NO), MHO (obesity defined as WC ≥80 cm in women and ≥90 cm in men), and MUO. We retained participants who were 50–85 years for avoiding differences in mortality for this reason of age. Likelihood ratio test was conducted to examine statistical interactions among BMI-MetS groups, sex, age (<65 years, ≥65 years), smoking status, drinking status, and physical activity in association with all-cause mortality by comparing –2 log likelihood χ^2^ between nested models, with or without the multiplication interaction terms.

All statistical analyses were conducted using SAS, version 9.4(SAS Institute Inc). Two-sided *P* < 0.05 was considered statistically significant.

### Study approval.

This study was approved by the ethics committees of Kailuan General Hospital and Beijing Tiantan Hospital, Capital Medical University. Written informed consent form was obtained from all participants.

## Author contributions

YW, WW, and HH conceived the study. AW, SC, and SW contributed population data resources. QT, AW, and YZ analyzed data. QT wrote the original draft. YW reviewed and edited manuscript.

## Supplementary Material

ICMJE disclosure forms

## Figures and Tables

**Table 2:**
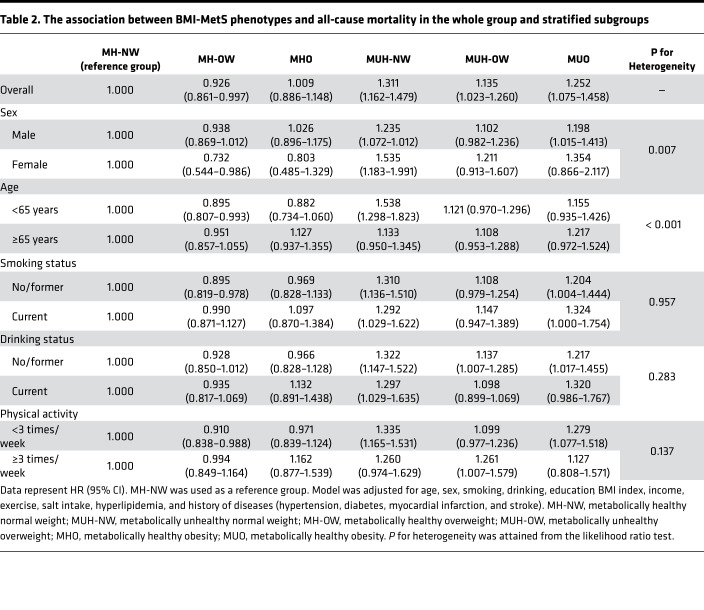
The association between BMI-MetS phenotypes and all-cause mortality in the whole group and stratified subgroups

**Table 1:**
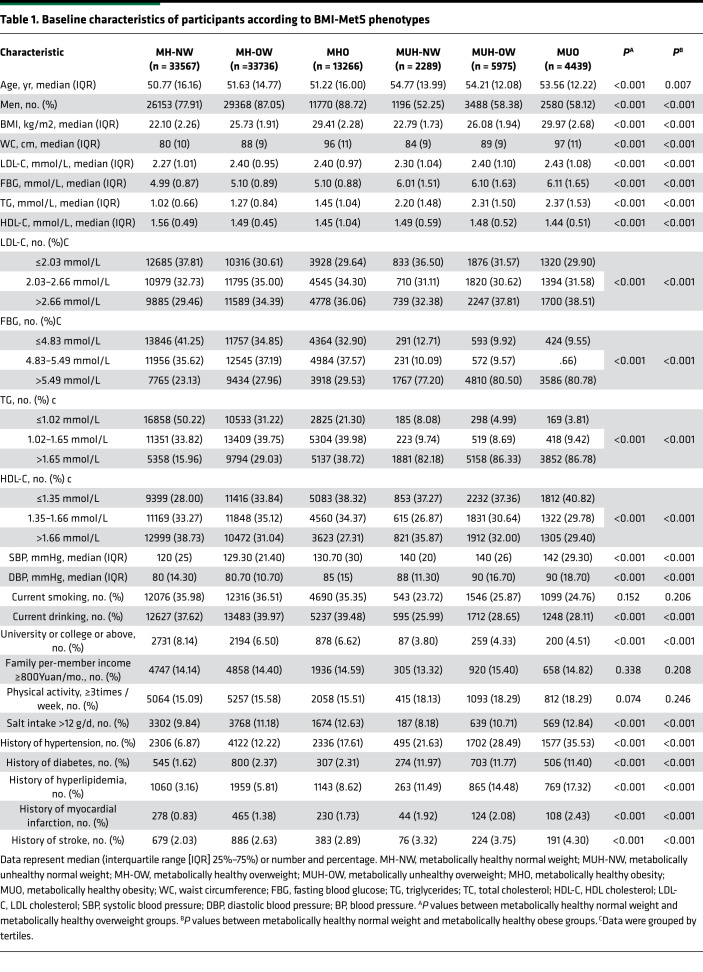
Baseline characteristics of participants according to BMI-MetS phenotypes

## Version 1 07/14/2020

In-Press Preview

## Version 2 08/20/2020

Electronic publication

## References

[1] Ng M (2014). Global, regional, and national prevalence of overweight and obesity in children and adults during 1980-2013: a systematic analysis for the Global Burden of Disease Study 2013. Lancet.

[2] NCD Risk Factor Collaboration (NCD-RisC) (2016). Trends in adult body-mass index in 200 countries from 1975 to 2014: a pooled analysis of 1698 population-based measurement studies with 19·2 million participants. Lancet.

[3] Hubert HB, Feinleib M, McNamara PM, Castelli WP (1983). Obesity as an independent risk factor for cardiovascular disease: a 26-year follow-up of participants in the Framingham Heart Study. Circulation.

[4] Rabkin SW, Mathewson FA, Hsu PH (1977). Relation of body weight to development of ischemic heart disease in a cohort of young North American men after a 26 year observation period: the Manitoba Study. Am J Cardiol.

[5] O’Brien EC, Fosbol EL, Peng SA, Alexander KP, Roe MT, Peterson ED (2014). Association of body mass index and long-term outcomes in older patients with non-ST-segment-elevation myocardial infarction: results from the CRUSADE Registry. Circ Cardiovasc Qual Outcomes.

[6] Yatsuya H (2014). Global trend in overweight and obesity and its association with cardiovascular disease incidence. Circ J.

[7] Riaz H (2018). Association between obesity and cardiovascular outcomes: a systematic review and meta-analysis of mendelian randomization studies. JAMA Netw Open.

[8] Arem H (2013). Prediagnosis body mass index, physical activity, and mortality in endometrial cancer patients. J Natl Cancer Inst.

[9] Ma J (2008). Prediagnostic body-mass index, plasma C-peptide concentration, and prostate cancer-specific mortality in men with prostate cancer: a long-term survival analysis. Lancet Oncol.

[10] Aune D (2016). BMI and all cause mortality: systematic review and non-linear dose-response meta-analysis of 230 cohort studies with 3.74 million deaths among 30.3 million participants. BMJ.

[11] Global BMI Mortality Collaboration null (2016). Body-mass index and all-cause mortality: individual-participant-data meta-analysis of 239 prospective studies in four continents. Lancet.

[12] Berrington de Gonzalez A (2010). Body-mass index and mortality among 1.46 million white adults. N Engl J Med.

[13] Sun YQ (2019). Body mass index and all cause mortality in HUNT and UK Biobank studies: linear and non-linear mendelian randomisation analyses. BMJ.

[14] Chen Y, Yang Y, Jiang H, Liang X, Wang Y, Lu W (2019). Associations of BMI and waist circumference with all-cause mortality: a 22-year cohort study. Obesity (Silver Spring).

[15] Bhaskaran K, Dos-Santos-Silva I, Leon DA, Douglas IJ, Smeeth L (2018). Association of BMI with overall and cause-specific mortality: a population-based cohort study of 3·6 million adults in the UK. Lancet Diabetes Endocrinol.

[16] Wang L (2016). Association of overweight and obesity with patient mortality after acute myocardial infarction: a meta-analysis of prospective studies. Int J Obes (Lond).

[17] Flegal KM, Kit BK, Orpana H, Graubard BI (2013). Association of all-cause mortality with overweight and obesity using standard body mass index categories: a systematic review and meta-analysis. JAMA.

[18] Whitlock G (2009). Body-mass index and cause-specific mortality in 900 000 adults: collaborative analyses of 57 prospective studies. Lancet.

[19] Kramer CK, Zinman B, Retnakaran R (2013). Are metabolically healthy overweight and obesity benign conditions?: A systematic review and meta-analysis. Ann Intern Med.

[20] Roberson LL (2014). Beyond BMI: The “metabolically healthy obese” phenotype & its association with clinical/subclinical cardiovascular disease and all-cause mortality -- a systematic review. BMC Public Health.

[21] Xu Y (2018). Association between the metabolically healthy obese phenotype and the risk of myocardial infarction: results from the Kailuan study. Eur J Endocrinol.

[22] Wei Y (2020). Metabolically healthy obesity increased diabetes incidence in a middle-aged and elderly Chinese population. Diabetes Metab Res Rev.

[23] Yeh TL, Chen HH, Tsai SY, Lin CY, Liu SJ, Chien KL (2019). The relationship between metabolically healthy obesity and the risk of cardiovascular disease: a systematic review and meta-analysis. J Clin Med.

[24] Xu R, Gao X, Wan Y, Fan Z (2019). Association of metabolically healthy overweight phenotype with abnormalities of glucose levels and blood pressure among Chinese adults. JAMA Netw Open.

[25] Kouvari M (2019). Transition from metabolically benign to metabolically unhealthy obesity and 10-year cardiovascular disease incidence: The ATTICA cohort study. Metabolism.

[26] Feng T (2019). Metabolically healthy obesity and risk for atrial fibrillation: the HUNT study. Obesity (Silver Spring).

[27] Hamer M, Stamatakis E (2012). Metabolically healthy obesity and risk of all-cause and cardiovascular disease mortality. J Clin Endocrinol Metab.

[28] Arnlov J, Ingelsson E, Sundstrom J, Lind L (2010). Impact of body mass index and the metabolic syndrome on the risk of cardiovascular disease and death in middle-aged men. Circulation.

[29] Appleton SL (2013). Diabetes and cardiovascular disease outcomes in the metabolically healthy obese phenotype: a cohort study. Diabetes Care.

[30] Sung KC (2015). All-cause and cardiovascular mortality among Koreans: effects of obesity and metabolic health. Am J Prev Med.

[31] Zheng R, Zhou D, Zhu Y (2016). The long-term prognosis of cardiovascular disease and all-cause mortality for metabolically healthy obesity: a systematic review and meta-analysis. J Epidemiol Community Health.

[32] Kuk JL, Rotondi M, Sui X, Blair SN, Ardern CI (2018). Individuals with obesity but no other metabolic risk factors are not at significantly elevated all-cause mortality risk in men and women. Clin Obes.

[33] Hamer M, Johnson W, Bell JA (2017). Improving risk estimates for metabolically healthy obesity and mortality using a refined healthy reference group. Eur J Endocrinol.

[34] Jae SY, Kurl S, Fernhall B, Kunutsor SK, Franklin BA, Laukkanen JA (2018). Are metabolically healthy overweight/obese men at increased risk of sudden cardiac death?. Mayo Clin Proc.

[35] Akinyemiju T (2018). A prospective study of obesity, metabolic health, and cancer mortality. Obesity (Silver Spring).

[36] Tian S, Xu Y, Dong H (2018). The effect of metabolic health and obesity phenotypes on risk of hypertension: A nationwide population-based study using 5 representative definitions of metabolic health. Medicine (Baltimore).

[37] Chang AR (2018). Metabolically healthy obesity and risk of kidney function decline. Obesity (Silver Spring).

[38] Lotta LA (2017). Integrative genomic analysis implicates limited peripheral adipose storage capacity in the pathogenesis of human insulin resistance. Nat Genet.

[39] Schulze MB (2019). Metabolic health in normal-weight and obese individuals. Diabetologia.

[40] Stefan N, Schick F, Haring HU (2017). Causes, characteristics, and consequences of metabolically unhealthy normal weight in humans. Cell Metab.

[41] Kwon BJ (2013). Metabolically obese status with normal weight is associated with both the prevalence and severity of angiographic coronary artery disease. Metabolism.

[42] Pajunen P (2011). Metabolically healthy and unhealthy obesity phenotypes in the general population: the FIN-D2D Survey. BMC Public Health.

[43] Hamer M, O’Donovan G, Stensel D, Stamatakis E (2017). Normal-weight central obesity and risk for mortality. Ann Intern Med.

[44] Aung K, Lorenzo C, Hinojosa MA, Haffner SM (2014). Risk of developing diabetes and cardiovascular disease in metabolically unhealthy normal-weight and metabolically healthy obese individuals. J Clin Endocrinol Metab.

[45] Eckel N, Muhlenbruch K, Meidtner K, Boeing H, Stefan N, Schulze MB (2015). Characterization of metabolically unhealthy normal-weight individuals: Risk factors and their associations with type 2 diabetes. Metabolism.

[46] Stefan N, Haring HU, Schulze MB (2018). Metabolically healthy obesity: the low-hanging fruit in obesity treatment?. Lancet Diabetes Endocrinol.

[47] Stefan N, Haring HU, Hu FB, Schulze MB (2013). Metabolically healthy obesity: epidemiology, mechanisms, and clinical implications. Lancet Diabetes Endocrinol.

[48] Wildman RP (2008). The obese without cardiometabolic risk factor clustering and the normal weight with cardiometabolic risk factor clustering: prevalence and correlates of 2 phenotypes among the US population (NHANES 1999-2004). Arch Intern Med.

[49] Suliga E, Ciesla E, Rebak D, Koziel D, Gluszek S (2018). Relationship between sitting time, physical activity, and metabolic syndrome among adults depending on body mass index (BMI). Med Sci Monit.

[50] Espinosa De Ycaza AE, Donegan D, Jensen MD (2018). Long-term metabolic risk for the metabolically healthy overweight/obese phenotype. Int J Obes (Lond).

[51] Eckel N, Li Y, Kuxhaus O, Stefan N, Hu FB, Schulze MB (2018). Transition from metabolic healthy to unhealthy phenotypes and association with cardiovascular disease risk across BMI categories in 90 257 women (the Nurses’ Health Study): 30 year follow-up from a prospective cohort study. Lancet Diabetes Endocrinol.

[52] Wang A (2013). Measures of adiposity and risk of stroke in China: a result from the Kailuan study. PLoS One.

[53] Wang A (2018). High SBP trajectories are associated with risk of all-cause death in general Chinese population. J Hypertens.

[54] Wu S (2012). Prevalence of ideal cardiovascular health and its relationship with the 4-year cardiovascular events in a northern Chinese industrial city. Circ Cardiovasc Qual Outcomes.

[55] Chen C, Lu FC, Department of Disease Control Ministry of Health PRC (2004). The guidelines for prevention and control of overweight and obesity in Chinese adults. Biomed Environ Sci.

[56] Alberti KG (2009). Harmonizing the metabolic syndrome: a joint interim statement of the International Diabetes Federation Task Force on Epidemiology and Prevention; National Heart, Lung, and Blood Institute; American Heart Association; World Heart Federation; International Atherosclerosis Society; and International Association for the Study of Obesity. Circulation.

[57] Wang A (2017). Changes in proteinuria and the risk of myocardial infarction in people with diabetes or pre-diabetes: a prospective cohort study. Cardiovasc Diabetol.

[58] Wang A (2017). Two-year changes in proteinuria and the risk of stroke in the Chinese population: a prospective cohort study. J Am Heart Assoc.

[59] Wang A (2014). Resting heart rate and risk of cardiovascular diseases and all-cause death: the Kailuan study. PLoS One.

